# Supplementary material for: Clinical, Histopathologic and Genetic Features of Rhabdoid Meningiomas
Source: Int J Mol Sci. 2023 Jan 6;24(2):1116. doi: 10.3390/ijms24021116 (PMC9865044; doi:10.3390/ijms24021116)
Supplement: Supplementary file 1 [file ijms-24-01116-s001.zip › 1_List of References to update.pdf]

58. Abolfotoh, M.; Al-Mefty, O.; Dunn, I.; Hong, C.; Lidov, H.; Tavanaiepour, D. Primary Calcified Rhabdoid Meningioma of the Cranio-Cervical Junction: A Case Report and Review of Literature. J. Craniovertebr. Junction Spine 2012, 3, 32, doi:10.4103/0974-8237.110127.
59. Ahmad, K.E.; Al-Jahdhami, S.; Ahmad, O. Rhabdoid Meningioma Presenting with Subependymal and Diffuse Meningeal Involvement but No Mass Lesion. J. Clin. Neurosci. 2010, 17, 1581–1582, doi:10.1016/j.jocn.2010.03.028.
60. Bannykh, S.I.; Perry, A.; Powell, H.C.; Hill, A.; Hansen, L.A. Malignant Rhabdoid Meningioma Arising in the Setting of Preexisting Ganglioglioma: A Diagnosis Supported by Fluorescence in Situ Hybridization. J. Neurosurg. 2002, 97, 1450–1455, doi:10.3171/jns.2002.97.6.1450.
61. Bansal, M.; Pathak, V.P.; Kishore, S.; Bansal, K.K. Rhabdoid Meningioma: Rapid Intraoperative Diagnosis on Squash Smears. Diagn. Cytopathol. 2009, NA--NA, doi:10.1002/dc.21276.
62. Batoroev, Y.K.; Nguyen, G.-K. Rhabdoid Meningioma Diagnosed by Imprint Cytology. Acta Cytol. 49, 464–465
63. Buccoliero, A.M.; Castiglione, F.; Degl'Innocenti, D.R.; Franchi, A.; Sanzo, M.; Cetica, V.; Giunti, L.; Sardi, I.; Mussa, F.; Giordano, F.; et al. Pediatric Rhabdoid Meningioma: A Morphological, Immunohistochemical, Ultrastructural and Molecular Case Study. Neuropathology 2011, 31, 59–65, doi:10.1111/j.1440-1789.2010.01113.x.
64. Cai, C.; Zhang, Q.; Shen, C.; Yang, W.; Hu, X.; Wang, C.; Ma, X.; Hou, Z. Rhabdoid Meningioma in a Child: Report of a Case and Literatures Review. Chinese J. Clin. Oncol. 2008, 5, 67–71, doi:10.1007/s11805-008-0067-6.
65. Chaturvedi, S.; Dua, R.; Singhal, S.; Kumari, R. Rhabdoid Meningioma with Cranial Nerve Involvement: Case Report of a Child. Clin. Neuropathol. 2008, 27, 248–251, doi:10.5414/NPP27248.
66. Costa Jr, L.B. da; Morais, J.V. de Meningioma Com Transformação Rabdóide: Relato de Caso. Arq. Neuropsiquiatr. 2003, 61, 277–280, doi:10.1590/s0004-282x2003000200023.
67. Dalal, V.; Siraj, F.; Kaur, M.; Shankar, K.B.; Singh, A. Bilateral Rhabdoid Meningioma Mimicking Glioma: An Unusual Occurrence. Ger. Med. Sci. 2017, 15, Doc12, doi:10.3205/000253.
68. Du, H.G.; Phuoc, V.X.; Hoang, N.D.; Tan, L.D.; Van Trung, N. Triad of Meningothelial Meningioma, Rhabdoid Meningioma and Ependymoma: Successful Management of an Extremely Rare Case. J. Surg. Case Reports 2020, 2020, doi:10.1093/jscr/rjaa267.
69. Dutta, D.; Lee, H.N.; Munshi, A.; Gupta, T.; Kane, S.; Sridhar, E.; Jalali, R. Intracerebral Cystic Rhabdoid Meningioma. J. Clin. Neurosci. 2009, 16, 1073–1074, doi:10.1016/j.jocn.2008.07.085.
70. Endo, K.; Tanaka, S.; Komagata, M.; Ikegami, H.; Kosaka, T.; Imakiire, A.; Serizawa, H. Rhabdoid Transformation of Recurrent Meningioma in the Cervical Cord: A Case Report. J. Orthop. Sci. 2004, 9, 323–326, doi:10.1007/s00776-004-0771-x.

71. Galloway, L.; Zilani, G.; Lammie, A.; Leach, P. Meningioma with Rhabdoid Features Combined with Meningioangiomatosis in Infancy: A Novel Combination. Child's Nerv. Syst. 2020, 36, 1311–1314, doi:10.1007/s00381-019-04486-5.
72. Jung Ho Han, Ho Jun Seol, Dong Gyu Kim, H.W.J. A Case of Rhabdoid Meningioma. J. Korean Neurosurg. Soc. 2006, 39(2), 144–147.
73. Hojo, H.; Abe, M. Rhabdoid Papillary Meningioma. Am. J. Surg. Pathol. 2001, 25, 964–969, doi:10.1097/00000478-200107000-00018.

74. Jun Jie, N.G.; Teo, K.A.; Shabbir, A.; Yeo, T.T. Widespread Intra-Abdominal Carcinomatosis from a Rhabdoid Meningioma after Placement of a Ventriculoperitoneal Shunt: A Case Report and Review of the Literature. Asian J. Neurosurg. 2018, 13, 176–183, doi:10.4103/1793-5482.181128.

75. Kashimura, H.; Ogasawara, K.; Mase, T.; Kurose, A. Unusual Growth Pattern of a Meningioma. Surg. Neurol. Int. 2012, 3, 63, doi:10.4103/2152-7806.97007.
76. Kirby, P.A. Rhabdoid Meningioma: Intraoperative Diagnosis Using Smear Preparation. Diagn. Cytopathol. 2003, 29, 292–296, doi:10.1002/dc.10375.
77. Koenig, M.A.; Geocadin, R.G.; Kulesza, P.; Olivi, A.; Brem, H. Rhabdoid Meningioma Occurring in an Unrelated Resection Cavity with Leptomeningeal Carcinomatosis. Case Report. J. Neurosurg. 2005, 102, 371–375, doi:10.3171/jns.2005.102.2.0371.
78. Mardi, K.; Thakur, R.C.; Biswas, B. Rhabdoid Meningioma Lacking Malignant Features: Report of a Rare Case with Review of Literature. Asian J. Neurosurg. 10, 172–174, doi:10.4103/1793-5482.153499.
79. Martínez-Lage, J.F.; Ferri Ñiguez, B.; Sola, J.; Pérez-Espejo, M.A.; Ros de San Pedro, J.; Fernandez-Cornejo, V. Rhabdoid Meningioma: A New Subtype of Malignant Meningioma Also Apt to Occur in Children. Child's Nerv. Syst. 2006, 22, 325–329, doi:10.1007/s00381-004-1094-0.
80. Martinez-Saez, E.; Malik, I.; Connor, S.; King, A. Combined Rhabdoid, Papillary and Adenocarcinomatous-like Elements in a Malignant Meningioma - a Potential Diagnostic Problem. Clin. Neuropathol. 31, 51–53, doi:10.5414/np300429.
81. Mathkour, M.; Garces, J.; Hanna, J.; Ware, M. Rare-Intracranial Atypical Rhabdoid Meningioma Lacking Malignant Features: Case Report and Literature Review. Neuro. Oncol. 2015, 17, v203.1-v203, doi:10.1093/neuonc/nov232.11.
82. McMaster, J.; Ng, T.; Dexter, M. Intraventricular Rhabdoid Meningioma. J. Clin. Neurosci. 2007, 14, 672–675, doi:10.1016/j.jocn.2006.02.019.
83. Morina, A.; Kelmendi, F.; Morina, O.; Pazanin, L.; Dragusha, S.; Ahmeti, F.; Morina, D. Rhabdoid Meningioma in an Eight-Year-Old Child. Med. Arh. 2010, 64, 123–124.
84. Nozza, P.; Raso, A.; Rossi, A.; Milanaccio, C.; Pezzolo, A.; Capra, V.; Gambini, C.; Pietsch, T. Rhabdoid Meningioma of the Tentorium with Expression of Desmin in a 12-Year-Old Turner Syndrome Patient. Acta Neuropathol. 2005, 110, 205–206, doi:10.1007/s00401-005-1048-y.
85. Parameshwaran Nair, R.; Vinod; Sarma, Y.; Nayal, B.; Kaur Dil, S.; Tripathi, P.K. Metastatic Rhabdoid Meningioma of the Parotid - Mimicking Primary Salivary Gland Neoplasm. Int. J. Surg. Case Rep. 2015, 6C, 104–106, doi:10.1016/j.ijscr.2014.10.048.
86. Parwani, A. V; Mikolaenko, I.; Eberhart, C.G.; Burger, P.C.; Rosenthal, D.L.; Ali, S.Z. Rhabdoid Meningioma: Cytopathologic Findings in Cerebrospinal Fluid. Diagn. Cytopathol. 2003, 29, 297–299, doi:10.1002/dc.10374.
87. Reddy, C.K.; Rao, A.D.; Ballal, C.K.; Chakraborti, S. Rhabdoid Meningioma: Report of Two Cases. J. Clin. Diagn. Res. 2015, 9, PD05-6, doi:10.7860/JCDR/2015/11163.5571.
88. Riqué Dormido, J.; Gómez Cárdenas, E.; Marín Láut, F.M.; Millan Ortega, I. Meningioma Intramedular Tipo Rabdoide. Reporte de Un Caso y Revisión de La Literatura. Neurocirugia 2019, 30, 202–205, doi:10.1016/j.neucir.2018.08.001.
89. Rittierodt, M.; Tschernig, T.; Samii, M.; Walter, G.F.; Stan, A.C. Evidence of Recurrent Atypical Meningioma with Rhabdoid Transformation and Expression of Pyrogenic Cytokines in a Child Presenting with a Marked Acute-Phase Response: Case Report and Review of the Literature. J. Neuroimmunol. 2001, 120, 129–137, doi:10.1016/S0165-5728(01)00425-8.

90. Santhosh, K.; Kesavadas, C.; Radhakrishnan, V. V; Thomas, B.; Kapilamoorthy, T.R.; Gupta, A.K. Rhabdoid and Papillary Meningioma with Leptomeningeal Dissemination. J. Neuroradiol. = J. Neuroradiol. 2008, 35, 236–239, doi:10.1016/j.neurad.2008.01.079.
91. Tian, Q.; Zhang, F.; Bi, L.; Wang, Y. Rhabdoid Meningioma: Analysis of One Case. Child's Nerv. Syst. 2014, 30, 189–191, doi:10.1007/s00381-013-2247-9.
92. Wakabayashi, K.; Suzuki, N.; Mori, F.; Kamada, M.; Hatanaka, M. Rhabdoid Cystic Papillary Meningioma with Diffuse Subarachnoid Dissemination. Acta Neuropathol. 2005, 110, 196–198, doi:10.1007/s00401-005-1037-1.
93. Xiao, G.-Q.; Burstein, D.E. Cytologic Findings of Rhabdoid Meningioma in Cerebrospinal Fluid. Acta Cytol. 2008, 52, 118–119, doi:10.1159/000325448.
94. Yeşiltaş, Y.S.; Gündüz, K.; Heper, A.O.; Erden, E. Ectopic Rhabdoid Meningioma of the Orbit in a Child: Case Report and Review of the Literature. J. Neurosurg. Pediatr. 2018, 22, 151–157, doi:10.3171/2018.1.PEDS17557.

95. Nilsen, G.; Liestøl, K.; Van Loo, P.; Moen Vollan, H.K.; Eide, M.B.; Rueda, O.M.; Chin, S.-F.; Russell, R.; Baumbusch, L.O.; Caldas, C.; et al. Copynumber: Efficient Algorithms for Single- and Multi-Track Copy Number Segmentation. BMC Genomics 2012, 13, 591, doi:10.1186/1471-2164-13-591.
96. Mermel, C.H.; Schumacher, S.E.; Hill, B.; Meyerson, M.L.; Beroukhim, R.; Getz, G. GISTIC2.0 Facilitates Sensitive and Confident Localization of the Targets of Focal Somatic Copy-Number Alteration in Human Cancers. Genome Biol. 2011, 12, R41, doi:10.1186/gb-2011-12-4-r41.
