# Supplementary material for: Clinical, Histopathologic and Genetic Features of Rhabdoid Meningiomas
Source: Int J Mol Sci. 2023 Jan 6;24(2):1116. doi: 10.3390/ijms24021116 (PMC9865044; doi:10.3390/ijms24021116)
Supplement: Supplementary file 1 [file ijms-24-01116-s001.zip › 2_Supplementary Matherial_IJMS_2023.pdf]

Supplementary Table S1. List of manuscripts reviewed for the identification and selection of RM tumors to be pooled for analysis in this study.

Type of study	Authors	Journal/year (Language other than English)	N. of RM included	PMID/DOI
Series (n=12)	Birua et al.$^{\$}$ [32]	Clin Neurol Neurosurg, 2021	11/137	34214868
	Kepes et al. [12]	Am J Surg Pathol, 1998	4	9500225
	Kim et al. [17]	AJNR, 2007	15	17846191
	Ko et al. [31]	J Clin Neurosci, 2007	16	17499508
	Kwon et al. [29]	Hum Pathol, 2013	30	22995327
	Perry et al. [13]	Am J Surg Pathol, 1998	15	9850174
	Shankar et al. [27]	Neuro Oncol, 2017	61	28170043
	Vaubel et al. [8]	J Neuropathol Exp Neurol, 2016	44 (160)*	26705409
	Wu et al. [5]	Neuropathol, 2011	6	21382093
	Wu et al. [14]	J Clin Neurosci, 2010	13	20537897
	Zhang et al. [9]	World Neurosurgery, 2018	11	29382616
	Zhou et al. [10]	World Neurosurg, 2013	11 (58)**	22902360
Case reports (n=63)	Abolfotoh et al. [58]	J Craniovertebr Junction Spine, 2012	1	23741128
	Ahmad et al. [59]	J Clin Neurosci, 2010	1	20800496
	Al-Habib et al. [20]	Clin Neuropathol, 2005	1	15696777
	Bannykh et al. [60]	J Neurosurg, 2002	1	12507148
	Bansal et al. [61]	Diagn Cytopathol, 2010	1	19941363
	Barresi & Caffo. [41]	J Neuropathol Exp Neurol, 2017	2	28860959
	Batoroev & Nguyen. [62]	Acta Cytol, 2005	1	16124182
	Buccoliero et al. [63]	Neuropathology, 2011	1	20408963
	Cai et al. [64]	Chin J Clin Oncol, 2008	1 (10)***	10.1007/s11805-008-0067-6
	Chaturvedi et al. [65]	Clin Neuropathol, 2008	1	18666441
	Christodoulides et al. [6]	World Neurosurg, 2020	1	31715408
	Cooper et al. [11]	Clinical neuropathology, 2004	2	14986929
	Costa Jr & Morais. [66]	Arq Neuropsiquiatr, 2003(Portuguese)	1	12806512
	Dalal et al. [67]	Ger Med Sci, 2017	1	28860959
	Du et al. [68]	J Surg Case Rep, 2020	1	32855801
	Dutta et al. [69]	J Clin Neurosci, 2009	1	18325590
	Jamal et al. [33]	Pediatr Dev Pathol, 2011	1	21417911
	Endo et al. [70]	J Orthop Sci, 2004	1	15168192
	Eom et al. [47]	Clin Neurol Neurosurg, 2009	1	19482417
	Galloway et al. [71]	Childs Nerv Syst, 2020	1	31897634
	Gayatri Rath et al. [39]	J Cancer Sci Ther, 2013	1	NA
	Hamilton et al. [16]	Brain Pathol, 2001	1	11556694
	Han et al. [72]	J Korean Neurosurg Soc, 2006	1	NA
	Hojo & Abe [73]	Am J Surg Pathol, 2001	1	11420471
	Hui et al. [45]	J Postgrad Med, 2015	1	25511215
	Jansen et al. [18]	Skull Base, 2003	1	15912159
	Jeong et al. [43]	Acta Cytol, 2013	1	24021412

Jun Jie et al. [74]	Asian J Neurosurg, 2018	1	29492156
Karabagli et al. [34]	Neuropathology, 2014	1	24702318
Kashimura et al. [75]	Surg Neurol Int, 2012	1	22754728
Kesavan. [15]	Singapore Med J, 2000	1	11193122
Khairy et al. [49]	World Neurosurg, 2019	1	31247353
Kirby. [76]	Diagn Cytopathol, 2003	1	14595799
Klein et al. [38]	Pathologe, 2002 (German)	1	12185783
Koenig et al. [77]	Diagn Cytopathol, 2003	1	15739568
Lofrese et al. [52]	Br J Neurosurg, 2011	1	21158508
Maiuri et al. [35]	Neurosurgical Review, 2021	1	34159472
Mardi et al. [78]	Asian J Neurosurg, 2015	1	153499
Martínez-Lage et al. [79]	Childs Nerv Syst, 2006	1	15800791
Martinez-Saez et al. [80]	Clin Neuropathol, 2012	1	22192705
Mathkour et al. [81]	Neuro Oncol, 2015	1	10.1093/neuonc/nov232.11
Matyja et al. [21]	Clin Neuropathol, 2010	2	20860894
Mawrin et al.[48]	Brain Pathol, 2006	1	15605995
McMaster et al. [82]	J Clin Neurosci, 2007	1	17433689
Mordechai et al. [50]	Pediatr Hematol Oncol, 2014	1	25116269
Morina et al. [83]	Med Arh, 2010	1	20514784
Motegi et al. [53]	Brain Tumor Pathol, 2012	1	22350616
Nozza et al. [84]	Acta Neuropathol, 2005	1	16025286
Parameshwaran Nair et al. [85]	Int J Surg Case Rep, 2015	1	25528037
Parwani et al. [86]	Diagn Cytopathol, 2003	1	14595800
Ravanpay et al. [28]	World Neurosurg, 2018	1	29981911
Reddy et al. [87]	J Clin Diagn Res, 2015	2	25859490
Riqué Dormido et al. [88]	Neurocirugía, 2019 (Spanish)	1	30219414
Rittierodt et al. [89]	J Neuroimmunol, 2001	1	10.1016/S0165-5728(01)00425-8
Rogerio et al. [4]	Clin Neuropathol, 2011	1	22011733
Saito et al. [42]	Brain Tumor Pathol, 2001	1	11908873
Santhosh et al. [90]	J Neuroradiol, 2008	1	18325590
Tian et al. [91]	Childs Nerv Syst, 2014	1	23974967
Wakabayashi et al. [92]	Acta Neuropathol, 2005	1	15981015
Wang et al. [51]	J Clin Neurosci, 2011	1	21349724
Xiao & Burstein. [93]	Acta Cytol, 2008	1	18323288
Yeşiltaş et al. [94]	J Neurosurg Pediatr, 2018	1	29726794

$: series excluded from the analyses because age and outcome data were out of the most frequently reported ranges; * manuscript reviewed 160 RM; **: this study includes 58 additional RM; ***this manuscript 10 reviewed additional RM; NA: not available. One additional RM publication by Kakar et al, 2016 IJNS (ISPUB.com) was not available in pubmed.

Supplementary Table S2. Series of meningiomas that contain WHO grade 3 RM (clinical data is not reported for every individual patient).

Clinical Data	RM studies*	Meningiomas with rhabdoid histology			N. of patients with WHO grade 3 RM (Sex)
		N. of patients (N. of samples)	Sex	Mean age (range)	
	2022. Actual series	23 (33)	9F/14M	61(34-84)	15 (5F/10M)
With individual information	2021 Birua G et al.[32] ϴ	11	5F/6M	34 (14-75)	5 (2F/3M)
	2018. Zhang et al.[9]	11	6F/5M	38 (4-63)	11 (6F/5M)
	2017. Shankar et al.[27]	14&47	34F/27M	56 (12-85)	1 (1F) &4 (2F/2M)
	2016. Vaubel et al.[8]	44	26F/18M	47 (9-79)	0
	2013. Zhou et al.[10]	12	6F/6M	44 (21-72)	12 (6F/6M)
	2011. Wu et al.[5]	6	3F/3M	45 (24-61)	6 (3F/3M)
	2010. Wu et al.[14]	13	6F/7M	50 (28-72)	4 (1F/3M)
	1998. Perry et al.[13]	15	7F/8M	52 (16-73)	8 (5F/3M)
	1998. Kepes et al.[12]	4	3F/1M	57 (44-84)	1 (1F)
Without individual information	2013. Kwon et al.[29]	30	6F/6M	52 (12-78)	30 (21F/9M)
	2007. Kim et al.[17]	15	11F/4M	52 (22-75)	NR
	2007. Ko et al.[31]	16	11F/5M	NR	NR

*: Year of publication and authors; ϴ: one series with mortality data out of usually reported range; F: female; M: male.

Supplementary Table S3. Overall clinical and histopathologic findings of 233 meningiomas with rhabdoid cell morphology reported previously in the literature, expressed as percentage and number of cases for each variable in brackets and the number of tumors with available clinical data for every variable in square brackets.

Clinical characteristics	Age group Adults	Children	Total	p value
	[N. of tumors with available data]			
Age (years)	[204/233] (88%)	[29/233] (12%)	[233]	
<5	-	17% (5)	2% (5)	0.000
6-10	-	28% (8)	3% (8)	
11-15	-	45% (13)	6% (13)	
16-18	-	10% (3)	1% (3)	
19-40	25% (50)	-	22% (50)	
41-60	49% (100)	-	43% (100)	
>60	26% (54)	-	23% (54)	
Sex	[204]	[29]	[233]	
Female	56% (114)	48% (14)	55% (128)	0.441
Male	44% (90)	52% (15)	45% (105)	
WHO grade	[194]	[26]	[220]	
WHO 1	30% (59)	4% (1)	27% (60)	0.10
WHO 2	39% (76)	50% (13)	41% (89)	
WHO 3	30% (59)	46% (12)	32% (71)	

Rhabdoid component		[171]	[23]	[194]	
	Pure rhabdoid	9% (15)	30% (7)	11% (22)	0.002
	Mixed histological patterns	91% (156)	70% (16)	89% (172)	
Mitotic rate		[144]	[19]	[163]	
	<4 mitoses	69% (100)	47% (9)	67% (109)	0.06
	>4-19 mitoses	225% (32)	47% (9)	25% (41)	
	≥20 mitoses	8% (12)	5% (1)	8% (13)	
Surgical resection		[131]	[28]	[159]	
	Total	66% (86)	68% (19)	66% (105)	0.823
	Partial	34% (45)	32% (9)	34% (54)	
Treatment		[167]	[29]	[196]	
No adjuvant treatment		50% (84)	28% (8)	47% (92)	0.009
	RDT	44% (74)	52% (15)	45% (89)	
	RDT+CMT	4% (6)	17% (5)	6% (11)	
	CMT	2% (3)	3% (1)	2% (4)	
Follow up (months)#	N. of cases (recurrence)	[83]	[19]	[102]	
	Median RFS (range)	34m (2m-171m)	38m (3m-132m)	35m (2m-171m)	
	No recurrence	61% (51)	42% (8)	58% (59)	0.124
	Recurrence	39% (32)	58% (11)	42% (43)	
	Median OS	[129]	[6]	[135]	
	Months (range)	47m (2m-191m)	64m (9m-204m)	48m (2m-204m)	
	≤12	11% (3)	16% (0)	11% (3)	
	13-36	27% (20)	0% (7)	27% (27)	0.514
	37-60	20% (11)	33% (2)	21% (13)	
	>60	40% (22)	50% (8)	41% (30)	
	Status	[127]	[6]	[133]	
	Alive	73% (93)	67% (4)	73% (97)	0.724
	Exitus	27% (34)	33% (2)	27% (36)	

RDT: radiotherapy; CMT: chemotherapy; RFS: recurrence fee-survival; OS: overall survival; #: only tumors with gross total resection were analyzed for RFS

Supplementary Table S4. Summary of clinicopathological characteristics and chromosomal alterations along the whole genome of our grade 3 RM tumors (24 arrays of 15 patients).

Tumor ID	Gender	Follow up			Rhabdoid component	Genetical profile	Nº of affected chr	Location of chromosomal CNAs
		Recurrence*	Exitus	Time[Δ]				
RM13	M	No	No	4	>50%	Losses	2	19, 22,
RM14	M	No	No	3	20-50%	Losses	3	19, 22, Y
RM11	M	No	Deceased	2.5	20-50%	Losses	3	14q, 19p, 22q
RM8	F	No	No	NA	>50%	Losses	5	2q, 6q, 8q, 19pq, 22
RM5	M	No	Deceased	NV[Θ]	>50%	Losses	6	1p, 6, 11p, 14, 22, Y
RM12[&]	M	No	Deceased	NV[ΘΘ]	>50%	Losses&Gains	3	3pq, 17q, Y
RM4	M	No	No	10	20-50%	Losses&Gains	7	1pq, 6, 8, 15, 19q, 20, 22
RM6_D_R1	M	1	No	7	20-50%	Losses&Gains	7	1pq, 6q, 9q,11p, 14, 18, 22
RM1	M	No	No	11	>50%	Losses&Gains	10	1p, 5, 10, 12, 13, 14, 17, 20, 22, Y
RM7_D_R1_R2_R3	M	3	No	13	20-50%	Losses&Gains	11/10/10/9	1pq, 6q, 8p, 10q, 12p, 14, 16, 18, 19p, 22, Y
RM2_D_R1	M	1	No	8	>50%	Losses&Gains	12	2, 5, 8, 9, 11, 12, 14, 15, 16, 17, 20, 22
RM10	F	No	Alive	4	>50%	Losses&Gains	13	3, 5, 7, 9, 10, 11, 14, 16, 17, 18, 20, 22, X
RM15	F	3	Deceased	11	20-50%	Losses&Gains	15	1pq, 4pq, 6q, 7pq, 8q, 9p, 10q, 12q, 14, 16q, 17q, 18, 19pq, 22, X
RM9_D_R1_R2_R3[$]	F	3	Deceased	12	>50%& 20-50%	Losses&Gains	16/14/13	1p, 2p, 3, 4, 6, 9q, 10, 11, 12p, 14, 15, 16q, 17p, 19q, 21, 22, Xq
RM3_D_R1_R2[$]	F	2	Deceased	13	>50%	Losses&Gains	10/16/19	1p, 2pq, 3pq, 4pq, 5, 6q, 7pq, 8, 9pq, 10pq, 11, 12, 15q, 16pq, 17q, 18q, 19p, 20q, 22q, X

F: female; M: male; *: recurrences expressed as number of relapses; Δ: follow-up time expressed in years; NA: not available; p or q: partial genetic losses or gains labeled with these letters indicate affected short or long chromosomal arm; [&]: multiple and spinal meningioma; D: tumor sample at diagnosis; R1: sample of first recurrence; R2: samples of second recurrence; R3: third relapse sample; [$]: different combination of affected chromosomes in diagnosis and recurrence samples, the chromosome changes are underlined; NV[Θ]: not valid for survival the patient died at 15 days after surgery; NV[ΘΘ]: patient died by a sepsis does not relate with the meningioma
